# Characteristics of older unpaid carers in England: a study of social patterning from the English Longitudinal Study of Ageing

**DOI:** 10.1093/ageing/afae049

**Published:** 2024-03-15

**Authors:** Laurie E Davies, Gemma F Spiers, David R Sinclair, Andrew Kingston, Barbara Hanratty

**Affiliations:** National Institute for Health and Care Research (NIHR) Policy Research Unit in Older People and Frailty / Healthy Ageing, Population Health Sciences Institute, Newcastle University, Newcastle-upon-Tyne, NE4 5PL, UK; National Institute for Health and Care Research (NIHR) Policy Research Unit in Older People and Frailty / Healthy Ageing, Population Health Sciences Institute, Newcastle University, Newcastle-upon-Tyne, NE4 5PL, UK; National Institute for Health and Care Research (NIHR) Policy Research Unit in Older People and Frailty / Healthy Ageing, Population Health Sciences Institute, Newcastle University, Newcastle-upon-Tyne, NE4 5PL, UK; National Institute for Health and Care Research (NIHR) Policy Research Unit in Older People and Frailty / Healthy Ageing, Population Health Sciences Institute, Newcastle University, Newcastle-upon-Tyne, NE4 5PL, UK; National Institute for Health and Care Research (NIHR) Policy Research Unit in Older People and Frailty / Healthy Ageing, Population Health Sciences Institute, Newcastle University, Newcastle-upon-Tyne, NE4 5PL, UK

**Keywords:** unpaid carers, older people, characteristics

## Abstract

**Background:**

A growing number of older people provide unpaid care, but contemporary research evidence on this group is limited.

**Aim:**

This study aims to describe the characteristics of older people who provide unpaid care and how these vary by socioeconomic position.

**Methods:**

Using recent information from the English Longitudinal Study of Ageing (ELSA wave 9, 2019), we analysed cross-sectional data on 1,282 unpaid carers aged ≥50. Data on sociodemographics, health, social wellbeing, care intensity and caregiver–recipient relationships were extracted. Total net non-pension wealth quintiles were used as a relative measure of socioeconomic position. Differences between the poorest and richest wealth quintiles were examined through logistic regression.

**Findings:**

Most older carers in ELSA were female and looking after another older person. Poor mental and physical health and social isolation were common, and socially patterned. Compared with carers in the middle wealth group, the poorest group were more likely to be living with the person they cared for (odds ratio (OR) 1.56 [95% confidence interval (CI) 1.03–2.36]) and more likely to experience loneliness (OR 2.29 [95% CI 1.42–3.69]), dependency (i.e. the need for help with activities of daily living) (OR 1.62 [95% CI 1.05–2.51]), chronic pain (OR 1.81 [95% CI 1.23–2.67]), a higher number of diseases (OR 1.75 [95% CI 1.15–2.65]) and fair/poor self-rated health (OR 2.59 [95% CI 1.79–3.76]). The poorest carers were also less likely to have a high quality of life (OR 0.51 [95% CI 0.33–0.80]) or be in work (OR 0.33 [95% CI 0.19–0.59]).

**Conclusion:**

Our findings suggest that financially disadvantaged unpaid carers (and their households) may have the greatest needs for intervention and support. Focussing resources on this group has potential to address social inequalities.

## Key Points

- The characteristics of unpaid older carers in England are socioeconomically patterned.
- Financially disadvantaged unpaid carers may have the greatest needs for intervention and support.
- Focussing resources on this group has potential to address social inequalities.

## Introduction

Unpaid or informal caregiving is critical to supporting individuals with health and social care needs [1], as high-income countries contend with how to finance care provision for growing older populations [2]. A large evidence base confirms that unpaid caring impacts numerous aspects of carers lives, including their health, social and financial wellbeing. However, our current understanding about the consequences of caring is limited in three ways [1, 3].

First, there is little evidence specifically about the demographics, health and wellbeing of *older* carers. Second, we know very little about how the patterns and consequences of caring vary by socioeconomic disadvantage. Finally, we do not yet have a clear understanding of the care needs of unpaid carers themselves. These are critical gaps given the expected rise in the number of older people providing care and the need to address health inequalities amongst carers.

Addressing these gaps will enhance our understanding of unpaid caring and inform policy efforts to developing meaningful support for carers. This research therefore aims to:

- Characterise the demographic profile, health, wellbeing and care needs of *older* unpaid carers.
- Explore if and how these characteristics of older carers are socioeconomically patterned.

## Methods

### Participants

We used data from the English Longitudinal Study of Ageing (ELSA), an ongoing population-based study of adults aged ≥50 in England that began in 2002 [4]. The original sample, derived from the Health Survey for England (HSE) that was itself designed to be nationally representative through stratified probability design, has been periodically refreshed (at waves 3, 4, 6, 7 and 9) with additional HSE sample members of particular age groups, to ensure that it remains representative of the older English population [5]. Using data from the most recent study wave (Wave 9, 2019) [6], 1,257 unpaid carers aged ≥50, with complete data for total net (non-pension) wealth, formed the basis for this analysis. We defined unpaid carers as an affirmative response to the question *‘Did you look after anyone in the past week […]? [By “look after” we mean the active provision of care?]’*, in accordance with previous research [7]. Our measure of socioeconomic status—total net non-pension wealth—reflects the current position and accumulation of assets over the life-course, and is believed to be a robust indicator of socioeconomic circumstance in studies of middle-aged and older people [8]. The variable represents the sum of net financial, physical and housing wealth, and full details of the wealth and debt components have been reported [9]. Wealth groups were defined as follows: first quintile (poorest, <£72 k), second-to-fourth quintile (middle, £72–646 k) and fifth quintile (richest, >£646 k) [10].

The remaining variables in our analysis were selected to describe participants’ sociodemographic characteristics, health, social wellbeing, and relationship to care recipient and intensity of supplied care (Supplementary data: Appendix 1).

### Statistical analysis

The characteristics of unpaid carers were examined through descriptive statistics. Socioeconomic differences in these characteristics were then assessed as follows (using wealth as the indicator and the middle wealth group as the reference): logistic regression (for age, sex, ethnicity, whether in work, whether working part-time, whether receiving carers allowance, relationship to care recipient, whether living with the care recipient, characteristics of the care recipient, disability, chronic pain, depression, social isolation and loneliness); ordinal logistic regression (for care intensity level, dependency, number of diseases, self-rated health, number of falls, anxiety and quality of life) and multinomial logistic regression (for marital status). Models were adjusted for age (as a continuous variable) and sex, and *P* <0.05 was regarded as statistically significant. Model assumptions were checked for the validity of results. Using the survey package [11] in R version 3.6.0, cross-sectional weights were applied to the analyses to mitigate sampling biases and non-response and ensure that the estimated parameters (effect sizes) from the models are a reflection of the demographic composition of England.

## Results

### Characteristics of unpaid carers

The weighted sample comprised 1,282 unpaid carers, of whom, most were women (60.1%, *n* = 770/1,282), aged ≥66 (57.6%, *n* = 739/1,282), white (93.8%, 1,202/1,282), married or cohabiting (77.8%, 997/1,282), not in work (58.7%, *n* = 752/1,282) and not in receipt of carers allowance (81.8%, *n* = 293/358), though there were limited data for this variable. In terms of relationships to care recipient and care intensity level, most looked after one person, including a spouse (30.7%, *n* = 393/1,282) or parent (24.5%, *n* = 314/1,282), did not live with the person they cared for (57.8% (*n* = 740/1,280)) and provided medium intensity care (65% (*n* = 822/1,265)). Of the carers providing help to persons who were frail/sick/disabled (57.9%, *n* = 742/1,282), the care recipients were usually aged >65 (81.4%, *n* = 604/742), and the care was provided at a low intensity level (70%, *n* = 420/601). In terms of the health characteristics of the unpaid carers, 11.4% (*n* = 133/1,161) had low dependency whereas 7.3% (*n* = 86/1161) had medium dependency; just over half experienced symptoms of depression (54.9%, *n* = 700/1,275); 41.5% (*n* = 528/1,273) reported conditions from two to three disease groups; 41.2% reported high anxiety (*n* = 480/1,165), and approximately one quarter had one or more falls in the past 2 years (25.3%, *n* = 197/779), chronic pain (26.2%, *n* = 335/1,280) and fair/poor self-rated health (24.6%, *n* = 316/1,282). In terms of social wellbeing, a majority of the unpaid carers experienced social isolation (73.3%, *n* = 592/808), whereas 18.5% felt lonely (*n* = 211/1,142), and just over a third had low quality of life (37.4%, *n* = 415/1,088) (Supplementary data: Appendix 2).

### Socioeconomic differences between unpaid carers

**Figure 1:**
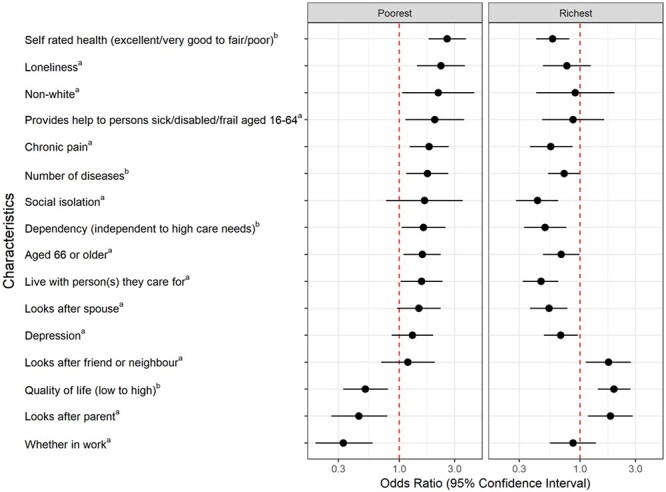
Characteristics *significantly associated* with unpaid carers in the poorest and richest wealth quintiles, compared with the middle group. ^a^Odds ratios correspond to the presence versus absence of the characteristic. ^b^Odds ratios correspond to per higher category of the characteristic.

Compared with unpaid carers in the middle wealth group, those in the poorest wealth quintile were more likely to report fair/poor self-rated health (odds ratio (OR) 2.59 [95% confidence interval (CI) 1.79–3.76]), loneliness (OR 2.29 [95% CI 1.42–3.69]), chronic pain (OR 1.81 [95% CI 1.23–2.67]), a higher number of diseases (OR 1.75 [95% CI 1.15–2.65]) and dependency (OR 1.62 [95% CI 1.05–2.51]). People in the poorest wealth quintile were also more likely to be aged ≥66 (OR 1.58 [95% CI 1.09–2.28]), non-white (OR 2.17 [95% CI 1.06–4.44]), live with the person they care for (OR 1.56 [95% CI 1.03–2.36]) and provide help to persons sick/disabled/frail aged 16–64 (OR 2.03 [95% CI 1.13–3.62]), but less likely to look after a parent (OR 0.45 [95% CI 0.26–0.79]), be in work (OR 0.33 [95% CI 0.19–0.59]) or have a high quality of life (OR 0.51 [95% CI 0.33–0.80]) (Figure 1).

Compared with those in the middle wealth group, people in the richest wealth quintile were less likely to report fair/poor self-rated health (OR 0.58 [95% CI 0.42–0.81]), chronic pain (OR 0.56 [95% CI 0.37–0.86]), a higher number of diseases (OR 0.73 [95% CI 0.53–0.99]), social isolation (OR 0.43 [95% CI 0.28–0.65]), dependency (OR 0.50 [95% CI 0.33–0.76]), older age (OR 0.69 [95% CI 0.48–0.99]) and depression (OR 0.68 [95% CI 0.49–0.96]). They were less likely to live with the person they care for (OR 0.46 [95% CI 0.32–0.65]) or look after a spouse (OR 0.54 [95% CI 0.37–0.78]), but more likely to look after a parent (OR 1.83 [95% CI 1.17–2.85]) or friend/neighbour (OR 1.76 [95% CI 1.12–2.74]) and have a high quality of life (OR 1.96 [95% CI 1.42–2.71]) (Figure 1).

**Figure 2:**
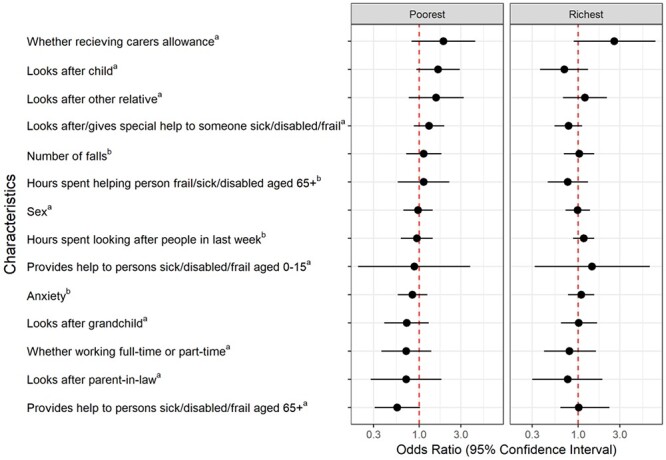
Characteristics *not* associated with unpaid caregivers in the poorest and richest wealth quintiles, compared with the middle group. ^a^Odds ratios correspond to the presence versus absence of the characteristic. ^b^Odds ratios correspond to per higher category of the characteristic.

Across the wealth quintiles, there was no significant difference in receipt of carers allowance; looking after a child/grandchild/parent-in-law or other relative; caring intensity; number of falls; sex; anxiety; whether working full-time or part-time, or the provision of help to persons sick/disabled/frail aged 0–15 or >65 (Figure 2).

## Discussion

This study examined the characteristics of unpaid older carers in the ELSA and found that outcomes are socioeconomically patterned.

Unpaid caring has been conceptualised as a social determinant of health as it places unpaid carers at greater risk of adverse outcomes. However, these have not been fully explored [3]. Our analysis finds that a substantial proportion of older unpaid carers in England experience chronic pain, poor self-rated health, multi-morbidity or dependency. Some older unpaid carers thus have care needs themselves, which may limit their ability to provide care, and to hence support the care recipient to ‘age in place’ [12]. Poor physical health, together with living and employment circumstances (being a co-resident carer or not being in work), may be associated with the poor mental health (e.g. depression) and social wellbeing (e.g. isolation, loneliness, quality of life) of unpaid carers in this analysis [13–17]. Socioeconomic differences were observed for all of these outcomes, with carers in the poorest wealth quintile having the worst experiences.

Previous research has stressed the importance of identifying groups vulnerable to the impact of unpaid caring [3]. Our analysis suggests that older carers in the poorest wealth quintile require more support. This group were far more likely to experience loneliness, which carries a number of health consequences [18], and has risen amongst unpaid carers since the COVID-19 pandemic [19]. Carers in the poorest wealth quintile were generally more likely to look after their spouse, or a frail/sick/disabled person aged 16–64. In contrast, carers in the richest wealth quintile were more likely to care for a parent. These differences are likely to reflect socioeconomic differences in life expectancy and the onset of ill health [20, 21]. Unpaid carers in the poorest wealth quintile were more likely to be aged over 66 [22] and less likely to report their ethnicity as white. This supports the limited UK research that has described clear variations in caregiving by ethnic group [23].

Some outcomes were common to unpaid carers irrespective of their socioeconomic position. For example, most carers in this analysis were women [24], across wealth quintiles. There were also no differences by wealth in hours spent caregiving or in the level of anxiety [25]. There were no differences in the proportion of people receiving carers allowance by wealth quintiles; however, sample numbers were small.

## Strengths and limitations

Carers in this study were drawn from a representative sample of people aged over 50. We were able to use information on wealth—one of the most appropriate measures of socioeconomic position in older people [8, 26–28]—and have described distinct patterns across social groups. We acknowledge the potential for unpaid carers to be under-represented in earlier ELSA study waves [29], and access to mortality data is restricted from wave 6 onwards. These factors led us to use data from the most recent study wave only (‘wave 9’). The cross-sectional nature of our analysis means that we have no information on duration of experiences for our study participants. Our analysis does not provide a complete picture of the consequences of unpaid caring as there are some outcomes that we could not measure, such as subjective carer burden and health service use. Furthermore, we had little information on the characteristics of the care recipients, and the type of care provided, including whether any of the unpaid carer participants were dementia caregivers or sandwich carers (i.e. those with the dual responsibility of caring for an older person as well as their dependent children), who are believed to experience higher rates of burden [30, 31]. In addition, only univariate associations were examined as this was a descriptive analysis, rather than an analysis for exploring a potential causal relationship between exposures and outcomes. Lastly, whilst our analysis focused on the consequences of caring in older carers, who are an under-researched group, we cannot assume that caregiving did not offer any rewards for them [32].

## Implications and conclusion

This research found a substantial proportion of older unpaid carers in England have complex health conditions and poor quality of life, particularly those who are financially disadvantaged. This is important as many carers struggle to access support, and feel overlooked by government as well as health and social care professionals [33, 34]. Our analysis thus extends the current knowledge base because it makes carers visible and emphasises caring as a social determinant of health. In terms of future directions, there is little evidence about what approaches and interventions work best to support the health of financially disadvantaged older carers. Long term, this risks widening health inequalities—not only amongst carers but amongst populations who are dependent on unpaid care to live well and with dignity. Future research could help to inform intervention strategies by providing a more in-depth and nuanced understanding of the unique needs and experiences of financially disadvantaged, older unpaid carers, and key areas of intersectionality. Such research should, for example, incorporate marginalised groups (such as ethnic minorities, and carers for people with substance use and poor mental health) and could seek to assess how disadvantaged caregivers have been affected by the uncertainty over anticipated social care reform. Promoting the health and wellbeing of older unpaid carers offers a potential pathway to ameliorating socioeconomic and age inequalities.

## Supplementary Material

aa-23-1708-File002_afae049

## Data Availability

The English Longitudinal Study of Ageing dataset is available in a public, open-access repository and can be accessed through the UK Data Service at https://ukdataservice.ac.uk/. The data can be used after registration and acceptance of end-user licence.
