# Supplementary material for: Characteristics of older unpaid carers in England: a study of social patterning from the English Longitudinal Study of Ageing
Source: Age Ageing. 2024 Mar 15;53(3):afae049. doi: 10.1093/ageing/afae049 (PMC10945290; doi:10.1093/ageing/afae049)
Supplement: aa-23-1708-File002_afae049 [file aa-23-1708-file002_afae049.docx]

**Characteristics of older unpaid carers in England: A study of social patterning from the English Longitudinal Study of Ageing**

**Supplementary data**

**Contents:**

- Appendix 1: Description of variables
- Appendix 2: Characteristics of unpaid carers

## Appendix 1: Description of variables

| **Variables** | **Description** |
| --- | --- |
| Age | To assess age differences, age was categorised as 50-65 and ≥66.^[35]^ |
| (i) Hours spent looking after people in the past week, and (ii) hours spent helping persons frail/sick/disabled aged 65+ | For both variables, the intensity of care supplied was described as low intensity (≤19 hours), medium intensity (20-49 hours) and high intensity (≥50 hours).^[36]^ |
| Number of diseases | Disease count was measured by response to ever having had any of the following self-reported doctor diagnoses: coronary heart disease (angina/myocardial infarction), stroke, hypertension, diabetes, arthritis, cancer, respiratory disease (asthma or chronic lung disease), depression, Alzheimer’s disease or dementia. |
| Dependency | Dependency of the person providing care (i.e. unpaid carers themselves) was categorised as independent (free from care), low (needs help less than daily), medium (needs help at regular times daily) or high (needs 24-h care).^[37]^ |
| Chronic pain | Using the self-reported answers to “Are you often troubled with pain?” and, if so, “How bad is the pain most of the time?” (with options of mild, moderate, or severe), we characterised chronic pain as pain that was classed as moderate or severe.^[38]^ |
| Quality of life | Quality of life was measured using the CASP-12 scale,^[39]^ and grouped into tertiles.^[40]^ |
| Self-rated health | Self-rated health was recoded as ‘Excellent/very good’, ‘good’, ‘fair/poor’. |
| Social isolation | Social isolation was defined as having a score of ≥2 from the following five indicators (with each having a score of 0 or 1):^[41]^   - Being unmarried/not co-habiting - Less than monthly contact with children (face-to-face, telephone, text/e-mail) - Less than monthly contact with other family (face-to-face, telephone, text/e-mail) - Less than monthly contact with friends (face-to-face, telephone, text/e-mail) - Not a member of any organisations, clubs or societies. |
| Loneliness | Loneliness was measured using the three-item University of California, Los Angeles (UCLA) Loneliness Scale.^[42]^ Participants were asked, on a three-point scale of ‘never or hardly ever’, ‘some of the time’ or ‘often’, how often they felt left out, isolated from others, or that they lack companionship. A score of six or more was classed as lonely. |
| Depression | Depressive symptoms were defined as an eight-item Center for Epidemiologic Studies Depression Scale score of ≥3.^[43]^ |
| Anxiety | Self-reported anxiety was measured using the Office for National Statistics anxiety scale, and categorised as very low (score of 0-1), low (score of 2-3), medium (score of 4-5) or high (score of 6-10).^[44]^ |

**References**

1. Petrillo M, Bennett M, G P. Cycles of caring: transitions in and out of unpaid care. London, 2022.
2. Office for National Statistics. 2011 Census analysis: Unpaid care in England and Wales, 2011 and comparison with 2001. 2013. <https://www.ons.gov.uk/peoplepopulationandcommunity/healthandsocialcare/healthcaresystem/articles/2011censusanalysisunpaidcareinenglandandwales2011andcomparisonwith2001/2013-02-15>.
3. Kingston A, Comas-Herrera A, Jagger C. Forecasting the care needs of the older population in England over the next 20 years: estimates from the Population Ageing and Care Simulation (PACSim) modelling study. The Lancet Public Health 2018; 3(9): e447-e55.
4. Steptoe A, Di Gessa G. Mental health and social interactions of older people with physical disabilities in England during the COVID-19 pandemic: a longitudinal cohort study. The Lancet Public Health 2021; 6(6): e365-e73.
5. Wiggins RD, Netuveli G, Hyde M, Higgs P, Blane D. The Evaluation of a Self-enumerated Scale of Quality of Life (CASP-19) in the Context of Research on Ageing: A Combination of Exploratory and Confirmatory Approaches. Social Indicators Research 2008; 89(1): 61-77.
6. Knesebeck OVD, Wahrendorf M, Hyde M, Siegrist J. Socio-economic position and quality of life among older people in 10 European countries: results of the SHARE study. Ageing & Society 2007; 27(2): 269-84.
7. Donovan NJ, Blazer D. Social Isolation and Loneliness in Older Adults: Review and Commentary of a National Academies Report. The American Journal of Geriatric Psychiatry 2020; 28(12): 1233-44.
8. Hughes ME, Waite LJ, Hawkley LC, Cacioppo JT. A Short Scale for Measuring Loneliness in Large Surveys: Results From Two Population-Based Studies. Research on Aging 2004; 26(6): 655-72.
9. Radloff LS. The CES-D Scale: A Self-Report Depression Scale for Research in the General Population. Applied Psychological Measurement 1977; 1(3): 385-401.
10. Office for National Statistics. Personal well-being in the UK: April 2021 to March 2022. 2022. <https://www.ons.gov.uk/peoplepopulationandcommunity/wellbeing/bulletins/measuringnationalwellbeing/april2021tomarch2022>

## Appendix 2: Characteristics of unpaid carers

|  | **All**  **(n=1282)** | **Men (n=512)** | **Women (=770)** | **P-value ^a^** |
| --- | --- | --- | --- | --- |
| **Socio-demographic characteristics** | | | | |
| Age  50-65  >66 | 42.3 (543)  57.6 (739) | 43.5 (222)  56.5 (289) | 41.7 (321)  58.3 (449) | 0.569 |
| Sex | 100.0 (1282) | 39.9 (512) | 60.1 (770) | - |
| Ethnicity  White  Non-white | 93.8 (1202)  6.2 (80) | 94.4 (483)  5.6 (29) | 93.4 (719)  6.6 (51) | 0.596 |
| Working status  Not in work  In work | 58.7 (752)  41.3 (530) | 58.4 (299)  41.6 (213) | 58.8 (453)  41.2 (317) | 0.905 |
| Full time or part time work (if in work)  Full time  Part time | 49.9 (263)  50.1 (264) | 71.0 (151)  29.0 (61) | 35.7 (112)  64.3 (202) | <0.001 |
| Whether receiving carers allowance  No  Yes | 81.8 (293)  18.2 (65) | 83.9 (138)  16.1 (27) | 80.0 (155)  20.0 (39) | 0.509 |
| Marital status  Married/Co-habiting  Single  Widowed  Divorced/separated | 77.8 (997)  7.1 (91)  6.0 (77)  9.1 (117) | 83.5 (427)  8.8 (45)  2.0 (10)  5.7 (29) | 73.9 (570)  6.0 (46)  8.6 (67)  11.5 (88) | <0.001 |
| **Relationship to care recipient and care level intensity** | | | | |
| Who they look after (multiple responses):  Spouse  Child  Grandchild  Parent  Parent-in-law  Other relative  Friend or neighbour | 30.7 (393)  11.8 (151)  18.3 (234)  24.5 (314)  5.8 (74)  7.5 (96)  11.9 (152) | 37.3 (191)  10.9 (56)  14.1 (72)  22.5 (115)  8.3 (42)  5.5 (28)  10.2 (52) | 26.2 (202)  12.4 (96)  21.0 (161)  25.8 (198)  4.0 (31)  8.8 (68)  13.0 (100) | <0.001  0.539  0.009  0.342  0.017  0.101  0.159 |
| Total number of people looked after (median, interquartile range) | 1 (1-2) | 1 (1-1) | 1 (1-2) | 0.277 ^b^ |
| Whether lives with person(s) they care for  Yes  No | 42.2 (540)  57.8 (740) | 48.2 (247)  51.8 (265) | 38.2 (293)  61.8 (475) | 0.003 |
| Hours spent looking after people in last week  Low intensity (≤19)  Medium intensity (20-49 hours)  High intensity (≥50 hours) | 20.8 (263)  65.0 (822)  14.2 (180) | 19.1 (96)  66.4 (334)  14.5 (73) | 21.9 (167)  64.0 (488)  14.1 (107) | 0.567 |
| **Caring for someone frail/sick/disabled** | | | | |
| Whether looks after or gives special help to someone sick/disabled/frail  Yes  No | 57.9 (742)  42.1 (540) | 61.0 (312)  39.0 (200) | 55.8 (430)  44.2 (340) | 0.125 |
| Number of people they provide this kind of help to (median, interquartile range) | 1 (1-1) | 1 (1-1) | 1 (1-1) | 0.943 ^b^ |
| Ages of people they provide this kind of help to (multiple responses):  0-15  16-64  65+ | 2.3 (17)  20.2 (150)  81.4 (604) | 2.8 (9)  21.4 (67)  79.9 (249) | 1.8 (8)  19.4 (84)  82.6 (355) | 0.422  0.602  0.447 |
| Hours spent helping person frail/sicked/disabled aged 65+  Low intensity (≤19 hours)  Medium intensity (20-49 hours)  High intensity (≥50 hours) | 70.0 (420)  11.5 (69)  18.7 (112) | 71.8 (177)  9.7 (24)  18.5 (46) | 68.6 (242)  12.7 (45)  18.7 (66) | 0.579 |
| **Health** | | | | |
| Dependency  Independent  Low  Medium  High | 80.2 (932)  11.4 (133)  7.3 (85)  0.9 (11) | 86.1 (414)  7.3 (35)  6.2 (30)  0.4 (2) | 76.1 (518)  14.3 (98)  8.1 (55)  1.4 (9) | <0.001 |
| Number of diseases  0  1  2-3  ≥4 | 18.0 (229)  32.2 (410)  41.5 (528)  8.3 (106) | 24.2 (123)  29.6 (151)  37.7 (192)  8.5 (44) | 13.8 (105)  34.0 (259)  44.1 (336)  8.1 (62) | <0.001 |
| Number of falls  0  1  2  ≥3 | 74.8 (583)  15.5 (121)  4.5 (35)  5.3 (41) | 75.5 (236)  14.9 (47)  5.0 (16)  4.5 (14) | 74.2 (347)  15.9 (74)  4.2 (19)  5.7 (26) | 0.807 |
| Chronic pain | 26.2 (335) | 21.2 (109) | 29.5 (227) | 0.005 |
| Depression | 54.9 (700) | 44.2 (226) | 62.1 (474) | <0.001 |
| Anxiety  Very low  Low  Medium  High | 21.8 (254)  23.7 (276)  13.3 (155)  41.2 (480) | 18.9 (91)  26.8 (129)  10.8 (52)  43.5 (209) | 23.9 (163)  21.5 (147)  15.0 (103)  39.6 (271) | 0.049 |
| Self-rated health  Excellent/very good  Good  Fair/poor | 41.5 (532)  33.9 (434)  24.6 (316) | 42.5 (218)  30.3 (155)  27.2 (139) | 40.9 (315)  36.2 (279)  22.9 (177) | 0.156 |
| **Social wellbeing** | | | | |
| Social isolation | 592 (73.3) | 70.0 (219) | 75.3 (372) | 0.197 |
| Loneliness | 18.5 (211) | 17.5 (83) | 19.2 (129) | 0.576 |
| Quality of life  Low  Medium  High | 37.4 (415)  30.2 (335)  30.5 (338) | 34.8 (157)  32.8 (148)  32.5 (147) | 39.3 (258)  28.5 (187)  32.2 (211) | 0.371 |

^a^ Chi-squared test, ^b^ Kruskal-Wallis test

Numbers are weighted and rounded
